# ACTH-independent macronodular adrenocortical hyperplasia reveals prevalent aberrant *in vivo* and *in vitro* responses to hormonal stimuli and coupling of arginine-vasopressin type 1a receptor to 11β-hydroxylase

**DOI:** 10.1186/1750-1172-8-142

**Published:** 2013-09-13

**Authors:** Johannes Hofland, Leo J Hofland, Peter M van Koetsveld, Jacobie Steenbergen, Wouter W de Herder, Casper H van Eijck, Ronald R de Krijger, Francien H van Nederveen, Maarten O van Aken, Johannes W de Groot, Thera P Links, Frank H de Jong, Richard A Feelders

**Affiliations:** 1 Department of Internal Medicine, Section of Endocrinology, P.O. Box 2040, Rotterdam, CA, 3000, The Netherlands; 2 Department of Surgery, Erasmus Medical Center, Rotterdam, The Netherlands; 3 Department of Pathology, Erasmus Medical Center, Rotterdam, The Netherlands; 4 Department of Pathology, Reinier de Graaf Gasthuis, Delft, The Netherlands; 5 Department of Internal Medicine, Haga Hospital, The Hague, The Netherlands; 6 Department of Internal Medicine, Isala Clinics, Zwolle, The Netherlands; 7 Department of Internal Medicine, University Medical Center Groningen, University of Groningen, Groningen, The Netherlands

**Keywords:** AIMAH, Cushing’s syndrome, Arginine-vasopressin

## Abstract

**Background:**

Adrenal Cushing’s syndrome caused by ACTH-independent macronodular adrenocortical hyperplasia (AIMAH) can be accompanied by aberrant responses to hormonal stimuli. We investigated the prevalence of adrenocortical reactions to these stimuli in a large cohort of AIMAH patients, both *in vivo* and *in vitro*.

**Methods:**

*In vivo* cortisol responses to hormonal stimuli were studied in 35 patients with ACTH-independent bilateral adrenal enlargement and (sub-)clinical hypercortisolism. *In vitro*, the effects of these stimuli on cortisol secretion and steroidogenic enzyme mRNA expression were evaluated in cultured AIMAH and other adrenocortical cells. Arginine-vasopressin (AVP) receptor mRNA levels were determined in the adrenal tissues.

**Results:**

Positive serum cortisol responses to stimuli were detected in 27/35 AIMAH patients tested, with multiple responses within individual patients occurring for up to four stimuli. AVP and metoclopramide were the most prevalent hormonal stimuli triggering positive responses *in vivo*. Catecholamines induced short-term cortisol production more often in AIMAH cultures compared to other adrenal cells. Short- and long-term incubation with AVP increased cortisol secretion in cultures of AIMAH cells. AVP also increased steroidogenic enzyme mRNA expression, among which an aberrant induction of *CYP11B1*. AVP type 1a receptor was the only AVPR expressed and levels were high in the AIMAH tissues. *AVPR1A* expression was related to the AVP-induced stimulation of *CYP11B1*.

**Conclusions:**

Multiple hormonal signals can simultaneously induce hypercortisolism in AIMAH. AVP is the most prevalent eutopic signal and expression of its type 1a receptor was aberrantly linked to *CYP11B1* expression.

## Background

Cushing’s syndrome (CS) can be divided into adrenocorticotropin (ACTH)-dependent and ACTH-independent disease [1]. The latter can in rare cases result from ACTH-independent macronodular adrenocortical hyperplasia (AIMAH). AIMAH is characterized by multiple bilateral nodules consisting of hyperplastic adrenal cells that lead to non-ACTH-dependent (over)production of cortisol.

The hypercortisolism in AIMAH patients is caused by an exaggerated or ectopic response to stimulation by hormonal signals. Receptors for these hormones, eutopically or ectopically expressed on adrenocortical cells and activated by endogenous hormones, stimulate intracellular pathways leading to (sub-)clinical CS [2]. Aberrant hormonal responses and the presence of hormone receptors in AIMAH have been well documented for glucose-dependent insulinotropic polypeptide (GIP) receptor [3-5], α4-, β1- and β2-adrenergic receptor (AR) [6,7], arginine-vasopressin (AVP) type 1a and 2 receptors (AVPR1A/2) [8,9], luteinizing hormone receptor (LHR) [10,11] and serotonin (5-HT) type 4 receptor (5-HT4R) [12,13]. Other possible aberrantly expressed receptors include the AngII type I receptor (AT1R) [14] and glucagon receptor [15,16].

Diagnostic protocols for AIMAH include administration of the various hormones or stimuli to patients [2]. The relevance of these effects has been shown by the results of administration of antagonists to the expressed receptors [6,10,17], confirmation of stimulatory effects of hormones on primary AIMAH cells *in vitro* and/or the detection of the hormone receptors on AIMAH cells. Furthermore, these effects have been described in adrenocortical adenomas and carcinomas, which can also overexpress receptors responsive to endocrine and/or paracrine signals [17].

Whereas the presence of aberrant receptors has been firmly established, little is known on the cause of such altered receptor expression and on downstream signals coupling receptor activation to stimulation of cell growth and steroidogenesis [18,19]. Although most patients present sporadically, some AIMAH cases occur in families [20], suggesting a possible genetic background. Investigations in murine models have shown that adrenal overexpression of GIP [21] and LH receptors [22] can lead to AIMAH and adrenal CS. This implies that the aberrant expression of these GPCRs may play a role in adrenal cell hyperplasia apart from modulating cortisol production. Most evidence on AIMAH pathophysiology in humans has been collected from case reports, small case series and reviews; only three centers have reported on larger groups of 16, 18 and 32 AIMAH patients, respectively [7,16,19]. Furthermore, no large series have systematically related clinical data to corresponding *in vitro* findings.

The current study evaluated the presence of aberrant hormonal responses in patients with AIMAH in order to discover novel pathways involved in the pathophysiology of this rare disease. We performed *in vivo* and *in vitro* stimulation tests with ACTH and multiple ligands for hormone receptors in the largest group of AIMAH patients described so far.

## Subjects and methods

### Patients

We included patients that presented with ACTH-independent bilateral adrenal enlargement with (sub-)clinical CS between 1994 and 2011. Clinical CS was defined by the presence of clinical symptoms and positive tests for hypercortisolism: the absence of a cortisol diurnal rhythm, increased 24h urinary free cortisol excretion, suppressed ACTH levels and/or failure to suppress cortisol levels below 50 nmol/l after 1mg dexamethasone overnight. Subclinical CS was defined by one or more positive tests for hypercortisolism in the absence of overt signs and symptoms of CS according to [23]. The study was approved by the medical ethical committee of the Erasmus MC and is in accordance with the use of residual tissues according to the Medical Research Involving Human Subjects Act.

All patients were admitted for measurement of baseline hormonal levels and subsequent administration of hormonal stimuli: 250 μg synacthen (Novartis, Basel, Switzerland) iv, 100 μg LH releasing hormone (LHRH, Ferring, Hoofddorp, The Netherlands) iv, 200 μg thyrotropin releasing hormone (TRH, Ferring) iv, a 2 h upright posture test, 10 mg metoclopramide (Pharmachemie, Haarlem, The Netherlands) orally, 1mg glucagon (Novo Nordisk, Alphen aan den Rijn, The Netherlands) iv, intravenous salt loading (NaCl 3% at 0.1 cc/kg/min) and a standard mixed meal (116 g carbohydrates, 27 g proteins, 14 g fat). From 2002 on, the intravenous salt loading test was replaced by intramuscular injection of 10 IU AVP (Ferring) [2]. ACTH levels were measured during the upright posture, intravenous salt loading and vasopressin tests to exclude effects of AVP on pituitary ACTH secretion. In case of clinical CS patients underwent bilateral laparoscopic adrenalectomy and were put on lifelong glucocorticoid and mineralocorticoid replacement.

### Tissue processing

Adrenal tissue was collected following adrenalectomy due to renal cell carcinoma (normal, n=3), AIMAH (n=22), ACTH-dependent hyperplasia (n=11), adrenocortical adenoma (n=11) or carcinoma (n=4). Parts of the tissue were stored at −80°C until RNA isolation. Other tissue parts were minced and transferred to a tube containing DMEM/F12 containing 5% fetal calf serum (FCS), penicillin and streptomycin (Invitrogen, Carlsbad, USA). Subsequently, tissues were dispersed into single primary adrenal cell suspensions using collagenase type I (Sigma-Aldrich, St. Louis, USA) as previously described [24]. Adrenal cell viability was checked with trypan blue and always exceeded 90%. 1,000,000 cells were put into 5 ml tubes for short-term incubations and, in case of sufficient cell yield, plated in 24 well plates at 100,000 cells per well for long-term culture.

### Short-term incubation

The cells were incubated in quadruplicate in a 2 ml volume containing 5% FCS with the following secretagogues: vehicle, ACTH, GIP (Sigma), metoclopramide, hCG (Organon, Oss, The Netherlands), epinephrine, norepinephrine (Centrafarm, Etten-Leur, The Netherlands), glucagon, AVP (Monarch Pharmaceuticals, Bristol, USA), desmopressin (Ferring), AngII (Sigma) or TSH (Genzyme, Naarden, The Netherlands). The PKA stimulator forskolin (FSK, Sigma) was added as a positive control. Secretagogues were selected on the basis of positive *in vivo* responses obtained in individual AIMAH patients. After the addition of hormones, tubes were incubated in a rocking water bath at 37°C for 2 h. Supernatants were removed and stored at −20°C until the measurement of cortisol.

### Long-term cell culture

Cells were allowed to attach overnight and the medium was changed to serum free DMEM/F12 the next day. After 24 h the hormonal stimuli also used for short-term incubation were added in quadruplicate and cells were cultured at 37°C for 48 h. Subsequently, supernatants were removed and stored at −20°C, whereas the attached cells were stored at −80°C until the isolation of RNA.

### Cortisol measurement and quantitative mRNA analysis

Cortisol levels were measured using a chemiluminescence-based method (Immulite, Siemens Diagnostics, Deerfield, USA; reference values for serum 200–800 nmol/l; for urine <850 nmol/24 h). RNA was isolated from plated cells and frozen adrenal tissue samples and reverse-transcribed as previously described [25]. The assays for the measurement of mRNA expression of the housekeeping gene *HPRT1*, the cholesterol transporter steroid acute regulatory protein (*STAR*) and steroidogenic enzymes (cytochrome P450 side chain cleavage [*CYP11A1*], 3β-hydroxysteroid dehydrogenase type 2 [*HSD3B2*]*,* 17-hydroxylase/17,20-lyase [*CYP17A1*]*,* 21-hydroxylase [*CYP21A2*] and 11β-hydroxylase [*CYP11B1*]) were as reported [26]. We used SYBR green-based assays for measurements of *AVPR1A* (F:TTTGTGATCGTGACGGCTTACA, R:GGTGATGGTAGGGTTTTCCGA) and *AVPR1B* (F:CAGCAGCATCAACACCATCT, R:CCATGTAGATCCAGGGGTTG) and purchased the *AVPR2* assay from Applied Biosystems (Hs00181055_m1). Positive controls for the assays for the vasopressin receptors consisted of human adrenal gland, kidney and pituitary gland, all obtained as residual tissues during surgical procedures within the Erasmus MC. Quantitative PCR was performed in a 12.5 μl mixture containing 20 ng cDNA [26]. PCR efficiency exceeded 90% for all assays used. mRNA levels were calculated relative to that of *HPRT1*, expression of which was shown to be stable under the conditions used, on the basis of the δCt-method.

### Data analysis and statistics

A full response was defined as a more than 50% increase of cortisol following the administration of the stimulus. Responses between 25-50% were considered as partial [2]. Group comparisons were made with Kruskal-Wallis followed by Dunn’s multiple comparison tests. Differences between 2 groups were analyzed by Mann–Whitney U or Wilcoxon signed rank tests for unpaired and paired observations, respectively. Spearman’s correlation coefficient was used for analysis of association between variables. Multiple testing was adjusted for by Bonferroni correction. All tests were calculated as two-tailed and a P-value below 0.05 was considered to be statistically significant.

## Results

### In vivo studies

Thirty-five AIMAH patients underwent *in vivo* evaluation of cortisol responses to eutopic and ectopic hormonal stimuli. Patient characteristics and basal hormone levels are summarized in Table 1 and Additional file 1: Table S1. All cases appeared to be sporadic; however familial screening was not performed. As expected, midnight serum cortisol levels, cortisol following dexamethasone overnight and 24 hour urinary free cortisol were all higher in patients with clinical compared to subclinical CS (all p<0.01, Table 1). Of note, in patients with subclinical CS urinary free cortisol levels were within the normal range whereas the dexamethasone suppression test was disturbed in the majority of patients. ACTH levels were suppressed in 39% of patients with subclinical CS. Adrenal diameters were larger in clinical CS patients than in subclinical CS (P<0.05, Table 1).

**Table 1:** **Characteristics of AIMAH patients evaluated *in vivo***

**Total**	**35**
Male/female	9/26
Age (years)	56.1±9.8
Adrenalectomy	22 (63%)
Hypercortisolism	
- SCS	13 (37%)
- CCS	22 (63%)
Diameter adrenal (mm)	
- left SCS	31.7±10.1
- left CCS	46.1±16.9*
- right SCS	27.2±8.8
- right CCS	43.6±17.0*
Hypertension	
- SCS	7 (54%)
- CCS	17 (77%)
Diabetes mellitus	
- SCS	6 (46%)
- CCS	6 (29%)
Bone loss	
- Osteopenia	
• SCS	4 (31%)
• CCS	4 (18%)
- Osteoporosis	
• SCS	5 (42%)
• CCS	5 (24%)
Midnight cortisol (nmol/l)	
- SCS	184±80
- CCS	319±145*
Urinary free cortisol (nmol/24h)	
- SCS	481±121 (0% > URL)
- CCS	1048±197* (37% > URL)
Cortisol after dexamethasone (nmol/l)	
- SCS	145±131 (92% > URL)
- CCS	296±183* (95% > URL)
ACTH (pmol/l)	
- SCS	1.47±0.92 (39% nd)
- CCS	0.85±0.89 (85% nd)

SCS: subclinical Cushing’s syndrome, CCS: clinical Cushing’s syndrome, URL: upper reference limit, nd: non-detectable, data presented as mean±SD, *P<0.05, compared to SCS. ^¶^ ACTH levels below the lower detection limit of 1.10 pmol/l were set at 0.55 pmol/l.

**Figure 1:**
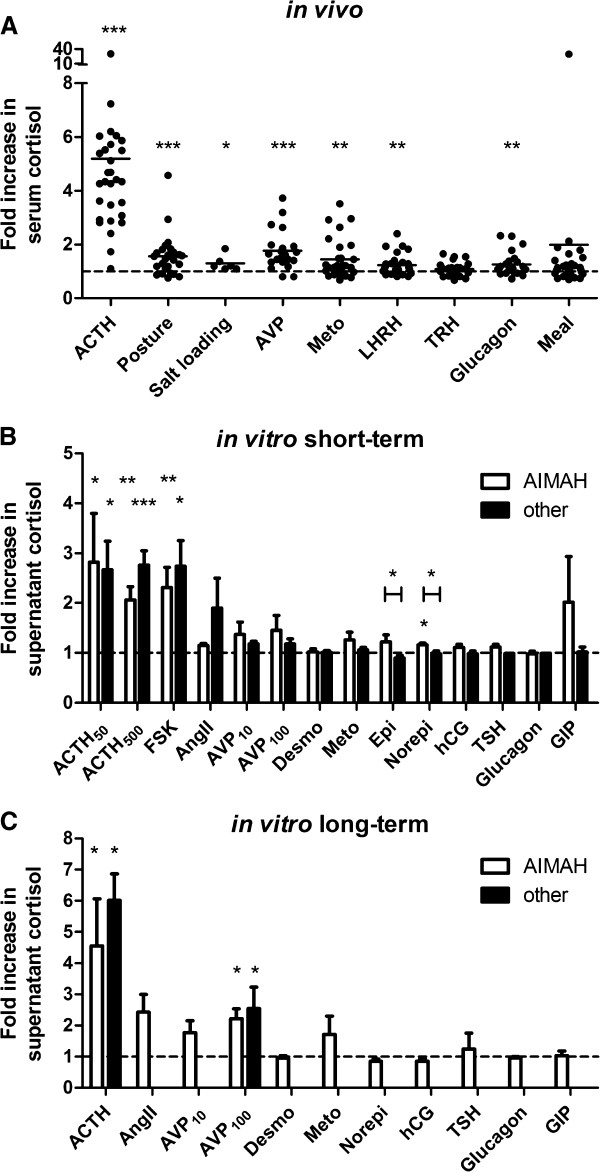
***In vivo *and *in vitro *induction of cortisol production following administration of hormonal stimuli. (A)** Patients were tested for the presence of aberrant expression of eutopic or ectopic hormone receptors by the administration of hormonal stimuli in various tests indicated on the X-axis. Line represents mean. *p<0.05, **p<0.01, ***p<0.001 compared to basal level (Wilcoxon signed rank test). **(B)** Primary cell cultures from AIMAH tissues (white bars) or from normal adrenals, ACTH-dependent adrenocortical hyperplasia or adenomas (other, black bars) were incubated for 2 h in the presence of specific hormone receptor agonists indicated on the X-axis. Concentrations used have been summarized in Table 3. Data are represented as mean+SEM. *p<0.05, **p<0.01, ***p<0.001 compared to vehicle control (Wilcoxon signed rank) or between pathological entities (Mann–Whitney U test). **(C)** Cortisol induction in media of AIMAH or other adrenal cells after 48 h incubation with specific hormone receptor agonists. Concentrations used are summarized in Table 4. Data are represented as mean+SEM. *p<0.05 compared to vehicle control (Wilcoxon signed rank). Numbers of patients are shown in Tables 2, 3 and 4.

ACTH administration increased serum cortisol 5.2±0.9-fold compared to baseline (mean±SEM, p<0.0001); positive responses were demonstrated in all but one patient. A single ACTH stimulation test in this patient with subclinical CS increased cortisol levels from 191 nM to only 211 nM. Of the additional hormonal stimuli tested, AVP gave the highest percentage of responses: 52% of patients displayed a >50% increase in cortisol levels. The other prevalent responses are also shown in Table 2 and the cortisol induction by these stimuli is indicated in Figure 1A. Overall, significant induction of cortisol was found for AVP, upright posture, metoclopramide, LHRH, glucagon and salt loading.

**Table 2:** ***In vivo *response to hormonal stimuli in patients with AIMAH**

**Stimulus**	**n tested**	**Partial response (25-50%) n (%)**	**Full response ≥50%) n (%)**
ACTH_1-24_	29	-	28 (97%)
Posture	26	4 (15%)	13 (50%)
Salt loading	6	1 (17%)	1 (17%)
AVP	21	6 (29%)	12 (52%)
Metoclopramide	29	3 (10%)	7 (24%)
LHRH	30	5 (17%)	5 (17%)
TRH	29	2 (7 %)	3 (10%)
Glucagon	26	4 (15%)	4 (15%)
Mixed meal	32	2 (6%)	6 (18%)
Number of aberrant responses		n	n
0		20	8
1		8	10
2		4	11
3		3	5
4		0	1

Twenty-seven (77%) patients showed a minimum of one full response to any administrated stimulus, apart from ACTH. One, 2, or 3 positive responses were demonstrated in 10, 11 and 5 patients, respectively. One patient had a positive response to 4 aberrant stimuli at *in vivo* testing (Table 2). Of the 26 patients that underwent an upright posture test and either salt loading or stimulation with AVP, 9 patients had discordant results, 5 (19%) of whom had only a complete response of serum cortisol to the upright posture test. Manipulation of angiotensin II or β-adrenergic receptors was not carried out in patients.

The cortisol responses to a standard mixed meal were inversely associated with the morning cortisol levels (r=−0.473, p=0.007) and correlated positively with the responses to ACTH (r=0.435, p=0.018). There were no significant differences in cortisol induction following the hormonal stimuli between patients with subclinical and clinical CS.

### Short-term in vitro studies

Cells for short-term (2 h) primary incubations were successfully obtained from the resected adrenal tissues in 14 out of 22 (64%) operated AIMAH patients (Table 3). Due to limited availability of cells, cultures could not be incubated with the complete panel of secretagogues (Additional file 1: Table S1). Incubation with ACTH (500 pg/ml) induced a full response in 60% of cultures. Augmented supernatant cortisol levels were found in 1 out of 4 cultures (25%) incubated with 100 nM AVP. In comparison, short-term metoclopramide incubation gave a full response in 2 out of 7 (29%) primary cultures. Norepinephrine, glucagon, desmopressin, hCG, angiotensin II and TSH did not augment cortisol secretion above 50% in any of the cultures investigated.

**Table 3:** **Two hour *in vitro *cortisol responses to hormonal stimuli in primary adrenal cell cultures**

		**AIMAH**				**Non-AIMAH**		
	**Dose**	**n**	**Partial response (%)**	**Full response (%)**	**Correlation with *in vivo *test**	**n**	**Partial response (%)**	**Full response (%)**
ACTH_1-24_	50 pg/ml	7	2 (29%)	4 (57%)	0.30	5	1 (20%)	4 (80%)
ACTH_1-24_	500 pg/ml	10	3 (30%)	6 (60%)	0.07	18	1 (6%)	16 (89%)
FSK	1 μM	8	0	7 (88%)	0.29	9	0	7 (78%)
AngII	10 nM	2	0	0	n.a. ^#^	2	1 (50%)	1 (50%)
AVP	10 nM	6	2 (33%)	1 (17%)	−0.72^†^; -0.50^#^	4	1 (25%)	0
AVP	100 nM	4	1 (25%)	1 (25%)	−1.00^†^; -0.80^#^	3	1 (33%)	0
Desmopressin	10 nM	5	0	0	−0.80	3	0	0
Metoclopramide	1 μM	7	0	2 (29%)	0.71	14	3 (21%)	0
Epinephrine	1 μM	7	1 (14%)	1 (14%)	−0.50^#^	5	0	0
Norepinephrine	1 μM	7	1 (14%)	0	−0.15^#^	5	0	0
hCG	100 IU/ml	10	3 (30%)	0	0.60	4	0	0
TSH	10 nM	4	0	0	−0.32	1	0	0
Glucagon	1 μM	4	0	0	0.50	1	0	0
GIP	100 nM	6	1 (17%)	1 (17%)	0.49^¶^	4	1 (25%)	0

^¶^ standard mixed meal, ^#^ upright posture test, ^†^ AVP im, n.a.: not applicable.

The mean cortisol induction was significant after 50 and 500 pg/ml ACTH (2.82±0.98-fold, p=0.018 and 2.06±0.27-fold, p=0.005), FSK (2.31±0.41-fold, p=0.005) and norepinephrine (1.16±0.04-fold, p=0.016), but not for the other stimuli (Figure 1B). Overall associations between hormonal effects obtained *in vivo* and *in vitro* were not significant for any of the stimuli investigated (Table 3). Similarly, tissues of patients who showed a complete response for a particular stimulus *in vivo* did not show higher cortisol levels after incubation with the hormonal equivalent compared to non-responders.

Short-term incubations were also performed with cells from 19 other adrenal tissues, consisting of 1 normal adrenal gland, 10 ACTH-dependent hyperplasias and 8 adrenocortical adenomas (Table 3 and Figure 1B). *In vivo* investigations were not available for these patients. *In vitro* responses to all individual stimuli were not significantly different between adrenal hyperplasia and adenoma samples. Full or partial cortisol responses were only observed after incubation with ACTH, FSK, AngII, AVP, metoclopramide and GIP. Only the mean cortisol responses to epinephrine and norepinephrine were significantly higher in AIMAH cultures than in the non-AIMAH cultures (P<0.05, Figure 1B).

### Long-term in vitro studies

Seven AIMAH tissues yielded sufficient cells to perform concurrent 48 h incubations with hormonal stimuli. The cortisol responses are shown in Table 4 and Additional file 1: Table S1. Again AVP (86%) and metoclopramide (50%) were the non-ACTH stimuli that most often led to >50% increases in cortisol levels. The mean induction of cortisol was only significant following the addition of ACTH (4.55±1.51-fold, p=0.016) and 100 nM AVP (2.21±0.32-fold, p=0.016), see Figure 1C; the sample sizes for the other groups were small. Again, there was a poor overall correlation between effects obtained *in vivo* and *in vitro* (Table 4). Cortisol levels did not increase after incubation of cells from the *in vivo* responders with the corresponding stimuli.

**Table 4:** **Forty-eight hour *in vitro *cortisol responses to hormonal stimuli in AIMAH cells**

	**Dose**	**n**	**Partial response n (%)**	**Full response n (%)**	**Correlation with *in vivo *test**	**Correlation with t=2 h**
ACTH_1-24_	10 ng/ml	7	0	6 (86%)	−0.39	0.20
AngII	100 nM	4	0	4 (100%)	1.00**^#^	n.a.
AVP	10 nM	3	0	2 (67%)	0.50^†^; n.a.^#^	n.a.
AVP	100 nM	7	1 (14%)	6 (86%)	−0.18^†^; -0.086^#^	−0.50
Desmopressin	10 nM	5	0	0	−0.67	1.00**
Metoclopramide	1 μM	4	0	2 (50%)	0.60	1.00**
Norepinephrine	1 μM	4	0	0	−0.50^#^	−0.50^#^
hCG	100 IU/ml	4	0	0	−0.60	−0.50
TSH	10 nM	2	0	1 (50%)	n.a.	n.a.
Glucagon	1 μM	4	0	0	−0.20	n.a.
GIP	100 nM	2	0	0	−1.00^¶^	n.a.

**p<0.01, ^¶^ standard mixed meal, ^#^ upright posture test, ^†^ AVP im, n.a. not applicable.

**Figure 2:**
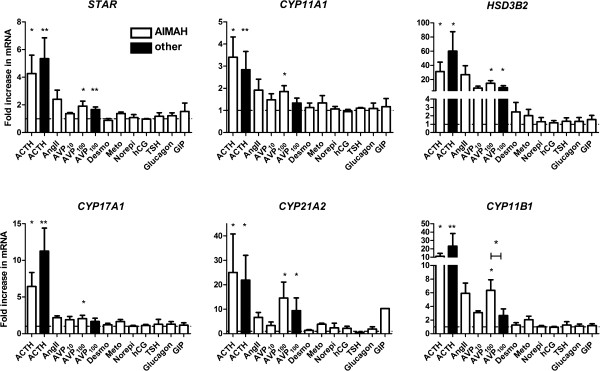
**Induction of *STAR *and steroidogenic enzyme mRNAs following addition of hormonal stimuli.** Primary cell cultures from AIMAH tissues (white bars) or from normal adrenals, ACTH-dependent adrenocortical hyperplasia, adenomas or carcinomas (other, black bars) were incubated for 48 hours in the presence of hormones depicted on X-axis. Concentrations used are summarized in Table 4. The mRNA levels of *STAR*, *CYP11A1*, *HSD3B2*, *CYP17A1*, *CYP21A2* and *CYP11B1* were studied by quantitative RT-PCR and calculated relative to *HPRT1* expression. Data are represented as mean+SEM. *p<0.05, **p<0.01, compared to basal level (Wilcoxon signed rank) or between pathological entities (Mann–Whitney U test).

We evaluated the effects of the hormonal stimuli on mRNA levels of *STAR* and the steroidogenic enzymes involved in cortisol synthesis (Figure 2). ACTH and AVP at 100 nM stimulated the expression of the cholesterol transporter and all steroidogenic enzymes studied in the AIMAH cultures. Although stimulatory effects were also found for the other secretagogues, these effects did not reach statistical significance, likely due to the small sample size (n≤4).

For this reason, we focused further on AVP and concurrently measured AVP effects in long-term adrenal cell cultures of non-AIMAH origin (i.e. 3 normal adrenals, 1 ACTH-dependent hyperplasia, 3 adenomas and 4 carcinomas). AVP at 100 nM significantly increased cortisol secretion in the 7 primary adrenal cell cultures with detectable supernatant cortisol levels (2.54±0.69-fold, p=0.016). AVP induced a >50% increase in cortisol levels in 71% of non-AIMAH adrenal cultures. The effects on steroidogenic enzyme mRNA expression after the addition of AVP were comparable between cultures from AIMAH cells and other adrenocortical tissues, with the exception of *CYP11B1* (Figure 2). 48 h culture in the presence of AVP stimulated *CYP11B1* expression 6.34±1.57-fold in AIMAH cells, compared to a 2.66±0.97-fold induction in cells of non-AIMAH adrenal origin (p=0.033). AVP-stimulated cortisol and *CYP11B1* levels were equal among the different groups of non-AIMAH tissues.

### AVP receptor expression

**Figure 3:**
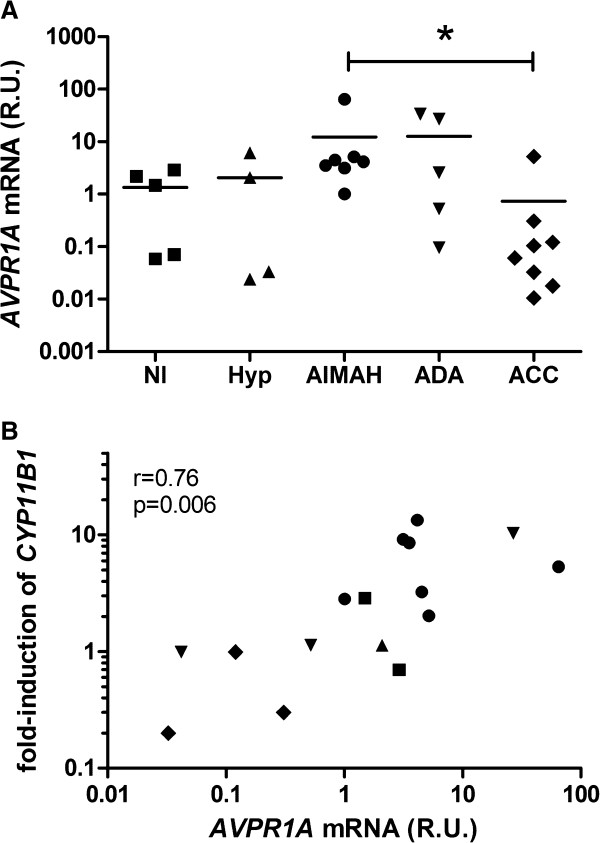
**AVP receptor expression in adrenal tissues. (A)** AVP type 1a receptor (V_1_, *AVPR1A*) mRNA expression in human adrenocortical tissues (Nl: normal, Hyp: ACTH-dependent hyperplasia, ADA: adrenocortical adenoma, ACC: adrenocortical carcinoma), measured by qRT-PCR. R.U.: relative units, compared to *HPRT1* expression. *p<0.05, Kruskal-Wallis test. V_2_ and V_3_ receptor expression levels were extremely low or undetectable. **(B)** Significant correlation between *AVPR1A* expression levels and the induction of *CYP11B1* by 100 nM AVP after 48 hours in cultures of primary cells of adrenal tissues. Corresponding icons for individual tissue groups are depicted in Figure A and B.

Expression of mRNA of vasopressin receptors (*AVPR1A*, *AVPR2*, *AVPR1B*) was studied in a panel of adrenocortical tissues, consisting of AIMAH, normal adrenals, ACTH-dependent hyperplasias, adenomas and carcinomas. The V_3_ (*AVPR1B*) was not detectable in any of the adrenocortical tissues studied, whereas V_2_ (*AVPR2*) mRNA was detectable in 10 out of the 29 samples, distributed among all groups, but at very low levels (Ct values>38). The ubiquitously expressed *AVPR1A* mRNA was found to be highest in the AIMAH samples. The expression levels in AIMAH were significantly increased compared to those in adrenocortical carcinomas (p=0.037, Figure 3A). AIMAH *AVPR1A* levels were not associated with clinical characteristics or the *in vivo* or *in vitro* cortisol induction following upright posture or AVP administration (p>0.05). On the other hand, *AVPR1A* levels in all tissues were significantly associated with the *in vitro* induction of *CYP11B1* by 100 nM AVP (r=0.76, p=0.006, Figure 3B).

## Discussion

AIMAH is a rare disease associated with the presence of aberrantly expressed eutopic and ectopic receptors on adrenocortical cells [2]. In the current study, ACTH still formed the most potent stimulus for steroidogenesis in AIMAH. ACTH stimulated cortisol production *in vivo* and *in vitro* in almost all patients. One patient showed only 10% increase in cortisol levels following ACTH. Unfortunately, repetitive testing was not available and incorrect administration or sample handling cannot be excluded in this case. The induction of cortisol by ACTH was comparable in primary cultures of AIMAH and non-AIMAH origin. We have previously demonstrated that MC2R levels, which are controlled by ACTH signaling through cAMP [27,28], are equal in AIMAH and other adrenal tissues [29]. Although plasma ACTH levels are low or undetectable in AIMAH patients, other GPCR-coupled pathways in AIMAH cells could stimulate cAMP formation and thereby ensure MC2R expression.

The most prevalent exaggerated responses to hormonal stimulation in AIMAH patients were to AVP and upright posture, confirming previous findings [16]. Several studies have now demonstrated the presence of the eutopically expressed V_1_ receptor in AIMAH tissues [9,30]. The (near) undetectable levels of V_2_ and V_3_ receptors combined with the absence of effects of desmopressin, a selective V_2_ agonist, plead against significant roles of these latter receptors in AIMAH. Although no overall significant differences in *AVPR1A* mRNA levels between AIMAH and other adrenal cells were detected [9,30], individual patients could still overexpress the V_1_ receptor [31-33]. AVP induced cortisol and mRNA expression of StAR and four steroidogenic enzymes uniformly in AIMAH and non-AIMAH cells, suggesting that AVP has a physiological effect on adrenocortical steroidogenesis. However, the selective stimulation of CYP11B1, a key enzyme in cortisol synthesis, in AIMAH by AVP indicates a novel molecular mechanism underlying the coupling between the V_1_ receptor and steroidogenesis in AIMAH. This suggestion is supported by the association between *AVPR1A* levels and the AVP-induced mRNA expression of *CYP11B1*. This correlation between receptor levels and cortisol stimulation is not present for MC2R and ACTH [29]. More efficient coupling of the V1 receptor to 11β-hydroxylase could form a cause of AIMAH that is characterized by normal GPCR levels. These findings in AIMAH might open up new opportunities for medical treatment with selective V_1_ receptor antagonists [34]. Adrenocortical carcinomas seem to have an impaired response to AVP, possibly due to decreased expression of the type V_1_ receptor.

The upright posture test was positive in half of the patients studied *in vivo*. Of the 13 patients with a positive response to upright posture, 8 reacted also positively to AVP administration. The other 5 patients could have reacted aberrantly to surges in catecholamines or AngII [2]. Interestingly, in the short-term experiments we found a difference in responsiveness to catecholamines between AIMAH and non-AIMAH cells. In contrast to the observation with AVP, this would suggest the presence of ectopic adrenergic receptors. Previous studies revealed that this could be related to adrenocortical expression of β_1_-, β_2_- or α_4_-adrenergic receptors [6,7].

The type 4 serotonin receptor (5HT4R) is expressed in the adrenal gland and its activation can affect cortisol production [35,36]. In the present study, 34% of patients had a >25% stimulation of serum cortisol levels following metoclopramide, an agonist of 5-HT4R, which was less than the 56% observed in the previous study [16]. *In vitro*, this response was also found in AIMAH samples as well as in controls. Moreover, there was no significant difference in response between AIMAH and non-AIMAH cells *in vitro*, making it questionable whether the response to 5-HT in AIMAH patients is truly aberrant.

Other hormonal stimuli can lead to a stimulation of serum cortisol in a minority of AIMAH cases. For LHRH, TRH, glucagon and GIP we have found that this is the case in 10-22% of patients. Adrenal LHR or GIPR expression was previously confirmed in 6 patients [5,11,33,37,38]. The association between cortisol induction following ACTH and after the standard mixed meal could be caused by food-induced stimulation of ACTH [39,40]. Unfortunately, we have no data on postprandial ACTH levels in these patients. The inverse correlation between morning cortisol and cortisol induction by a mixed meal reflects higher cortisol increments in patients with low basal cortisol levels. The cause of this finding is unknown, but might reflect higher adrenal sensitivity to GIP at lower cortisol levels. *In vitro*, we found no major effects of the other hormonal stimuli on cortisol production besides in individual cultures.

The clinical description of AIMAH cases has often been coupled with *in vitro* investigations on patient tissue samples. Several AIMAH patients with discrepancies between clinical and experimental hormonal responses have been described [41,42], which is in contrast to the majority of AIMAH studies in which identical *in vitro* and *in vivo* hormonal effects are obtained (reviewed in [2]). We have evaluated *in vitro* responses in 17 primary cultures of AIMAH tissues and detected a poor overall correlation between clinical and experimental responses to individual stimuli. Possible causes include publication bias of those patients in whom effects could be replicated *in vitro*, hormonal effects through the pituitary, the concentrations of stimuli used *in vitro* or the experimental set-up. Since ACTH levels were currently only measured in the *in vivo* response tests relating to AVP, effects of the other hormonal stimuli through pituitary secretion of ACTH cannot be excluded and might also cause dissociation between clinical and experimental effects. With respect to the latter cause, we also found clear differences and an overall lack of association between short-term and long-term effects of the stimuli on cortisol concentrations. These conclusions should however be drawn with caution due to the small sample sizes of cultures in some of the short- and long-term experiments. Besides the rarity of the disease, the low percentage of patients being operated because of clinical disease and the multitude of testable hormonal stimuli hamper large *in vitro* studies with all possible secretagogues in AIMAH patients. Also in this study limited availability of cells did not allow for systematic *in vitro* evaluation of all possible stimuli.

Responses to the aberrant hormonal stimuli were not associated with *in vivo* hormonal activity since patients with clinical and subclinical CS showed comparable results to stimuli. It should be emphasized that the definition of subclinical CS is controversial as some clinical symptoms of CS can be recognized in patients with subtle cortisol overproduction. In addition, no cut-off values of diagnostic tests have been established that define subclinical hypercortisolism. The findings on *in vivo* responsiveness to hormonal stimuli are a confirmation of an earlier study [16], in which it was postulated that clinical and subclinical CS represent a continuum of disease rather than two separate entities. This hypothesis is supported by the smaller adrenal sizes detected in subclinical CS patients; these patients could progress into clinical CS when adrenal size and coupled steroidogenic capacity increase.

Exaggerated responses to hormonal stimuli have been reported for multiple cases and case series in adrenal hyperplasia and adenomas, although the responses in healthy individuals have not been investigated for all hormones. It is therefore uncertain to what extent *in vivo* responses are the results of aberrant expression patterns of hormone receptors. Previous [36,43] and current *in vitro* data obtained in the non-AIMAH tissues suggest significant effects of AVP and possibly 5-HT. In our analysis we found no differences in responsiveness to hormonal stimuli among ACTH-dependent hyperplasia, adrenal adenoma and carcinoma samples, although numbers were small. Systematic *in vivo* comparisons for responses between healthy individuals and AIMAH patients might improve the definition of an aberrant response. The arbitrarily used criterion of >50% elevation in serum cortisol may also be modified if effects in healthy individuals would be identified.

## Conclusions

Multiple hormonal responses frequently occur in AIMAH patients, with AVP and 5-HT most commonly triggering aberrant eutopic responses. AVP induces steroidogenic enzyme expression in both AIMAH and non-AIMAH adrenocortical tissues. However, in AIMAH there appears to be an aberrant coupling of normal levels of *AVPR1A* expression to the induction of *CYP11B1* expression that may be involved in the pathogenesis of AVP-mediated cortisol overproduction. This may provide an opportunity for medical treatment, targeting the AVP type 1a receptor in a subset of AIMAH patients. Catecholamines appear to represent the most prevalent *in vitro* ectopic response in AIMAH patients.

## Competing interests

The authors declare that there is no conflict of interest that would prejudice the impartiality of this scientific work.

## Authors’ contributions

JH, LJH, PMvK & JS performed the experiments. JH, LJH, PMvK, RRdK, FHvN, CvE, WWdH, MvA, JWdG, TPL and RAF were involved in patient care and/or tissue collection. JH, LJH, WWdH, FHdJ and RAF designed the study. All authors had the opportunity to revise the manuscript and agree to its final content.

## Supplementary Material

Additional file 1 Results of clinical and experimental hormonal stimuli on cortisol induction in individual AIMAH patients. Click here for file
